# Defeat and entrapment: more than meets the eye? Applying network analysis to estimate dimensions of highly correlated constructs

**DOI:** 10.1186/s12874-018-0470-5

**Published:** 2018-01-25

**Authors:** Thomas Forkmann, Tobias Teismann, Jana-Sophie Stenzel, Heide Glaesmer, Derek de Beurs

**Affiliations:** 1 Institute of Medical Psychology and Medical Sociology, University Hospital of RWTH Aachen University, Pauwelsstraße 19, 52074 Aachen, Germany; 2 Department of Clinical Psychology and Psychotherapy, Ruhr-Universität Bochum, Bochum, Germany; 3 Department of Medical Psychology and Medical Sociology, University of Leipzig, Leipzig, Germany; 4 NIVEL, Netherlands Institute for Health Services Research, Utrecht, the Netherlands

**Keywords:** Defeat, Entrapment, Network analysis, Correlation, Suicide

## Abstract

**Background:**

Defeat and entrapment have been shown to be of central relevance to the development of different disorders. However, it remains unclear whether they represent two distinct constructs or one overall latent variable. One reason for the unclarity is that traditional factor analytic techniques have trouble estimating the right number of clusters in highly correlated data. In this study, we applied a novel approach based on network analysis that can deal with correlated data to establish whether defeat and entrapment are best thought of as one or multiple constructs.

**Methods:**

Explanatory graph analysis was used to estimate the number of dimensions within the 32 items that make up the defeat and entrapment scales in two samples: an online community sample of 480 participants, and a clinical sample of 147 inpatients admitted to a psychiatric hospital after a suicidal attempt or severe suicidal crisis. Confirmatory Factor analysis (CFA) was used to test whether the proposed structure fits the data.

**Results:**

In both samples, bootstrapped exploratory graph analysis suggested that the defeat and entrapment items belonged to different dimensions. Within the entrapment items, two separate dimensions were detected, labelled internal and external entrapment. Defeat appeared to be multifaceted only in the online sample. When comparing the CFA outcomes of the one, two, three and four factor models, the one factor model was preferred.

**Conclusions:**

Defeat and entrapment can be viewed as distinct, yet, highly associated constructs. Thus, although replication is needed, results are in line with theories differentiating between these two constructs.

## Background

Gilbert and Allan [1] proposed two central constructs that they assumed to be involved in the development of depressive disorders: defeat and entrapment. Experiences of *defeat* have been described as the perception of a failed struggle, feelings of powerlessness and a sense of losing social status or missing personal goals [2]. According to Gilbert and Allan [1], feelings of *entrapment* occur when people are motivated to escape threat or a stressful, unpleasant state or situation but the flight is blocked because of internal (e.g., insufficient coping agency, severe health problems or feelings of guilt) or external circumstances (e.g. no help by others, problems at work, school or in personal relations) [1, 3, 4].

In recent years, research showed the transdiagnostic relevance of these constructs in the development of depressive, anxiety, and post-traumatic stress disorders (PTSD), as well as suicidality [5, 6]. Moreover, Griffiths et al. [7] presented evidence in line with the assumption that defeat and entrapment precede the development of depression and anxiety in a longitudinal research design [8]. Furthermore, defeat and entrapment play a crucial role in theories on the development of suicidal ideation and behavior [9–11]. Recently, the Integrative Motivational-Volitional Model of Suicidal Behavior (IMV, [12, 13]) was introduced that assumes that suicidal ideation and behavior develop, if people encounter defeating experiences or situations and then cannot escape from these situations and experiences, thus feeling entrapped. A burgeoning literature reports results that are in line with the central assumptions of the IMV-model (e.g. [14–16]).

### Assessment of defeat and entrapment

Defeat and entrapment are usually assessed with the Defeat Scale (DS) and the Entrapment Scale (ES), both developed by Gilbert and Allan [1]. The two scales consist of 16 items each using a five-point Likert scale. The DS showed good internal consistency and good convergent and criterion validity in terms of positive relations with depression, hopelessness and suicidality in students, patients, and male prison inmate samples [1, 6, 17–22]. The ES showed comparable psychometric characteristics in terms of internal consistency and convergent and criterion validity [1, 23]. The ES was originally designed as a two-dimensional instrument, distinguishing between internal and external entrapment [1]. Internal entrapment is measured with six items (e.g., “I would like to escape from my thoughts and feelings” or “I feel trapped inside myself”) and external entrapment with ten items (e.g. “I have a strong desire to escape from things in my life” or “I can see no way out of my current situation”). Gilbert and Allan [1] reported a correlation between both scales of *r* = .75 but nonetheless argue that they are differentiable facets of the construct. However, subsequent research confirmed the close relation between internal and external entrapment and suggested that entrapment should be best conceptualized as a unidimensional construct [24].

Moreover, a vivid debate is ongoing about the factorial validity of the defeat and entrapment scale. While the two instruments were originally designed and applied as being two separate scales, recent research suggests that they appear to represent the same construct [7, 24, 25]. This implies that the constructs themselves might not be distinct but rather two sides of the same story [24]. Consequently, Griffiths et al. [26] developed the short defeat and entrapment scale (SDES). The SDES consists of eight items, four indicating defeat and four entrapment. The eight items of the SDES were chosen from the 32 items of the original DS and ES by means of a principal-axis exploratory factor analysis (EFA): Griffiths et al. [26] picked the four highest loading items of the DS and the four highest loading items of the ES from the EFA based on data of *N* = 262 participants from the community. The authors presented then a series of analyses supporting unidimensionality, internal consistency, and validity of this set of eight items building the SDES.

However, within the field of psychometrics, it is known that well established analytic techniques such as exploratory factor analysis (EFA) and Confirmatory Factor Analysis (CFA) might underestimate the number of factors when factors are highly correlated [27]. When Griffith et al. [26] tested a two-factor model of defeat and entrapment, they found that it actually fitted the data better than a one factor model. Yet, because the two factors were highly correlated (*r* = 0.91), they decided to stay with the one factor model. Within this article, we applied a novel technique based on network modelling that has been found to outperform traditional techniques when data are highly correlated. We will compare the number of identified clusters with the results from standard factor techniques based on data from an online and a clinical sample, and discuss its scientific and clinical relevance.

### Network analysis

In the past years, there has been a specific interest in the estimation of network models using psychological data [28]. Network analysis allows to visualize and estimate the association between variables, without assuming any underlying dimensional structure a priori [29]. A network consists of nodes (the items) and the pairwise relation between the items (edges). When two items have a pairwise interaction after conditioning for all other items in the dataset (so-called partial correlation), they are connected via a line (edge). Importantly, the interpretability of a network is highly increased by applying a penalized maximum likelihood estimation called LASSO. After applying LASSO estimation on the partial correlation matrix, non-relevant spurious partial correlations are set to zero, resulting in a network of direct non-spurious relations between nodes [30]. Network modelling has successfully been applied within for example the field of depression research (e.g., [31]), PTSD (e.g., [32]), and recently, suicidology (e.g., [33]).

### Identifying dimensions within a network

Although network analysis does not assume any underlying latent structure a priori, researchers and clinicians are still interested in the clustering of nodes. Indeed, Golino and Epskamp [27] argue that clusters in a network are similar to latent variables. The LASSO estimations result in a sparse matrix that is better attuned to identifying clusters in highly correlated data. Next, an algorithm called walktrap can be used to identify numbers of clusters or latent variables within this sparse matrix [29]. In simulation studies, the application of a walktrap algorithm on a sparse matrix was found to outperform traditional methods like parallel analysis and eigenvalue decomposition when analyzing data with multiple strongly correlated latent factors [27]. Confirmatory Factor Analysis can then be applied to test whether the proposed structure fits the data.

### Study aim

The aim of the present study was to estimate the number of dimensions within a network of the 32 items of the DS and ES in an online and a clinical sample using graph techniques and to compare the results with exploratory factor analysis.

## Methods

### Online sample

Between December 2015 and April 2016, data was collected through an anonymous online survey using the SoSci-server [30]. The study was approved by the Ethics Committee of the Ruhr-Universität Bochum, Germany. Participants were recruited through postings at two universities (Aachen and Bochum) and several psychotherapy outpatient units as well as social media (e.g., Facebook). When surfing on the study website, before the start of the survey, participants were informed about the purpose and content of the survey and provided with useful addresses (i.e., telephone numbers of helplines and contact information for therapy institutions), in case they felt burdened due to the content of the study or had suicidal thoughts in general. They were also provided with contact addresses in case they had any questions. In order to take part in the study, participants had to be at least 18 years old and give their consent to participation at the beginning of the study. Participants could only proceed to the next questionnaire, if they had answered the previous questionnaire completely. It was possible to either take a break (and possibly continue to fill out the rest of the survey later on) or to stop the survey completely (and therefore delete all data) at every time during the survey.

At the end of the study, participants had the opportunity to take part in a raffle, where – as an incentive for participation – five Amazon gift cards each valued at 15 euros were raffled. On average, participants spent 15.3 min filling in the questionnaires.

### Clinical sample

Data of 147 patients who answered the 32 items on defeat and entrapment within 2 weeks after being admitted to German psychiatric hospitals after a suicide attempt or a severe suicidal crisis were used to verify the number of dimensions of the defeat and entrapment scale in a clinical sample. Participants were approached personally while in hospital, informed about the study aims and procedures and gave written informed consent prior to participation. Patients were eligible to participate if they were at least 18 years old, capable to read and communicate in German language and were not suffering from a psychotic disorder or acute substance intoxication. They filled in the ES and DS in a paper-pencil-version together with additional questionnaires reported elsewhere. This is preliminary data that was collected within the scope of a larger ongoing research project called “PRESS: Prediction of the longitudinal development of suicidal thoughts and behaviors - a validation of the interpersonal theory of suicidal behavior” which was approved by the ethics committees of the Ruhr-Universität Bochum, Germany, the Medical Faculty of the RWTH Aachen University, Germany, and the Medical Faculty of the University of Leipzig, Germany.

### Instruments

#### German version of the defeat scale (DS)

The defeat scale was originally developed by Gilbert and Allan [1] and consists of 16 items (three inversely coded). Participants are asked to rate how strongly they agree with each of the 16 items on a five-point scale from “never” (0) to “always” (4) regarding the last week (e.g. ‘I feel defeated by life’; ‘I feel down and out’). Higher scores indicate higher feelings of defeat. Previous studies found high internal consistency (a = .86, [1, 17, 18, 21]). The German version used in this study [34] has been translated according to the guidelines of the ISPOR Task Force for Translation and Cultural Adaption and shows good internal consistency (Cronbach‘s *α* = 0.95) [35].

#### German version of the entrapment scale (ES)

The ntrapment scale developed by Gilbert and Allan [1] measures feelings of entrapment, i.e., the impression of wanting to escape a burdensome situation/state of mind, but not being able to do so. Participants are supposed to indicate how strongly they agree with each of the 16 items of the instrument when thinking about the past week (e.g. ‘I feel powerless to change myself’; ‘I am in a relationship I can’t get out of’). All items are rated on a five-point scale ranging from “not at all” (0) to “very much” (4). In a validation study of the German version that was used in the current study, Trachsel et al. [23] demonstrated high internal consistency (*α* = .95; *N* = 540).

#### Assessment of suicidality and depression

In the online sample, suicidal ideation was assessed using the Depressive Symptom Inventory – Suicidality Subscale (DSI-SS) [36] ; German version: [37]) consisting of four items designed to measure the intensity of suicidal ideation symptoms over the past 2 weeks. The sum-score ranges from 0 to 12. The internal consistency for the DSI-SS in the online sample was α = .93. In the clinical sample, the Beck Scale for Suicide Ideation (BSS) ([38]; German version: [39]) consisting of 21 items was used. The sum-score ranges from 0 to 42. The internal consistency in the clinical sample was α = .87. Depressive symptoms over the past 2 weeks were assessed in both samples using the Rasch-based depression screening [40] consisting of 10 items. Sum-scores >11 are indicative for a potential depressive episode (Cronbach’s α = .92 (clinical sample) and α = .94 (online sample)).

### Data analysis

Descriptive analyses were done using PASW Statistics 20.0.0.

All other analyses were performed with R version 3.3.3. [41].

#### Exploratory and confirmatory analysis

First, we conducted an exploratory factor analysis (EFA) to uncover the underlying structure of the items, and estimate item loadings. Next, we plotted the eigenvalues to the number of factors to see where the slope is leveling off (the elbow criterion within a scree plot). Next, we applied a confirmatory factor analysis (CFA) , using the WLSMV estimater to estimate the model parameters to test whether the proposed factor structure fits the data. EFA was estimated using the psych package for R [42], CFA was estimated using LAVAAN for R [43].

#### Explanatory graph analysis (EGA)

Exploratory Graph Analysis is a novel method to find dimensions in sparse networks [27]. Using the regularized partial correlation matrix, a gaussian graphical model is estimated. Next, the walktrap algorithm from the Igraph package is used to detect the number of dense subgraphs within the Gaussian graphical model. The *walktrap* algorithm provides a measure of similarities between vertices (i.e. nodes where two or more edges meet) based on random walks which can capture the community/cluster structure in a graph. In order to group the vertices or nodes, the distance between nodes is computed. If the distance between two nodes is large they belong to a different community and if the distance is small, they are grouped in the same community [44].

**Fig. 1:**
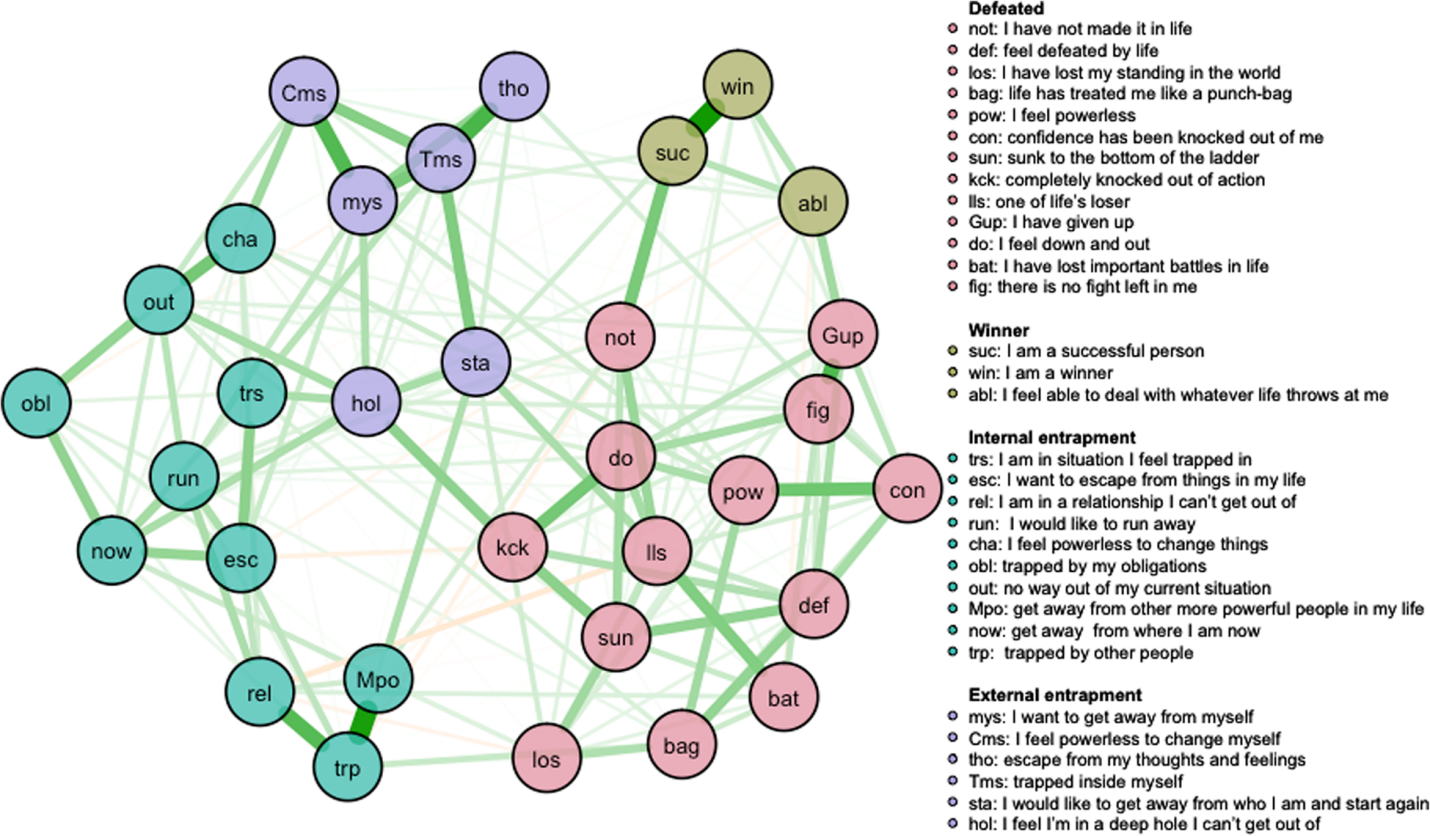
Network analysis of the online sample: coloring indicates clustering as identified with the walktrap algorithm. *Note:* the cluster labelled “winner” consists of the three defeat items that are positively framed

The intuition behind a random walk is the following: random walks on a graph (such as in Fig. 1) are likely to get “stuck” within highly correlated parts of that graph, corresponding to clusters or dimensions within the network. For more technical details we refer to [44]. To obtain a more stable solution, we re-estimated the number of dimensions of 1000 bootstraps from the sample using a parametric approach. The median community solution is then returned. We tested the fit of the proposed factor solution to the data with a CFA, and compared the outcome with the CFA of the EFA solution.

## Results

*N* = 480 (74% female; M_age_ = 28.5, SD_age_ = 11.1, Range: 18–80 years) participated in the online survey. *N* = 142 (29.6%) participants of the online sample reported some amount of suicidal ideation indexed by a DSI-SS sumscore ≥ 1. The clinical sample consisted of *N* = 147 patients (52% female; M_age_ = 36.0, SD_age_ = 12.7, Range: 18–68 years) who completed all items. *N* = 111 (75.5%) patients of the clinical sample reported at least one life time suicide attempts. Descriptive information of the two samples is presented in Table 1.

**Table 1:** Descriptive information for the online and the clinical sample

		Online sample (*N* = 480)			Clinical Sample (*N* = 147)	
		N	%		N	%
Gender (female)		355	74		78	53
		M	SD		M	SD
Age		28.50	11.10		36.00	12.70
Depression		8.35	8.41		27.57	8.98
Suicidality	DSI-SS	5.00	1.94	BSS	19.45	7.14

### Results in the online sample (*N* = 480)

#### Exploratory factor analysis (EFA)

**Fig. 2:**
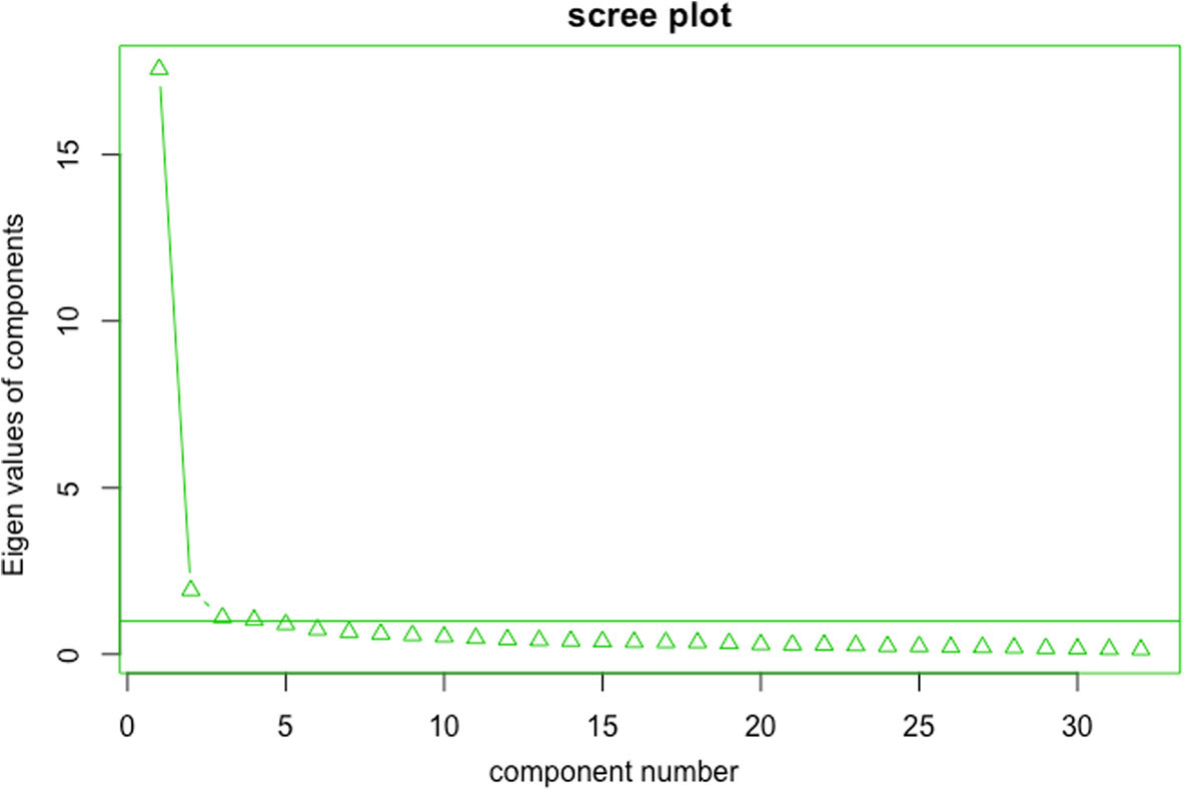
Scree plot of the online sample (*n* = 480)

Within the online sample, EFA indicated that 53% of the variance was explained by one factor. The scree plot also suggested that a one factor solution fits the data best (Fig. 2). Within the one factor model, all items loaded above 0.4 except item 32 (0.39), indicating all items load at least reasonably well on the factor (Table 2), with many items loading > 0.6.

**Table 2:** Factor loadings for a single factor. (d) is item from the defeat scale, (e) is item from the entrapment scale

Items of the defeat and entrapment scale	Online sample	Clinical sample
1. I feel that I have not made it in life (d)	0.72	0.70
2. I feel that I am a successful person. (d)	0.63	0.61
3. I feel defeated by life. (d)	0.80	0.76
4. I feel that I am basically a winner. (d)	0.55	0.63
5. I feel that I have lost my standing in the world. (d)	0.72	0.64
6. I feel that life has treated me like a punch-bag. (d)	0.70	0.67
7. I feel powerless. (d)	0.75	0.83
8. I feel that my confidence has been knocked out of me. (d)	0.76	0.73
9. I feel able to deal with whatever life throws at me. (d)	0.59	0.70
10. I feel that I have sunk to the bottom of the ladder. (d)	0.78	0.75
11. I feel completely knocked out of action. (d)	0.75	0.80
12. I feel that I am one of life’s losers. (d)	0.77	0.79
13. I feel that I have given up. (d)	0.80	0.76
14. I feel down and out. (d)	0.81	0.84
15. I feel that I have lost important battles in life. (d)	0.66	0.71
16. I feel that there is no fight left in me. (d)	0.79	0.76
17. I am in situation I feel trapped in. (e)	0.75	0.64
18. I have a strong desire to escape from things in my life. (e)	0.67	0.63
19. I am in a relationship I can’t get out of. (e)	0.78	0.52
20. I often have the feeling that I would just like to run away. (e)	0.75	0.61
21. I feel powerless to change things. (e)	0.59	0.68
22. I feel trapped by my obligations. (e)	0.80	0.57
23. I can see no way out of my current situation. (e)	0.55	0.66
24. I would like to get away from other more powerful people in my life. (e)	0.78	0.59
25. I have a strong desire to get away and stay away from where I am now. (e)	0.56	0.43
26. I feel trapped by other people. (e)	0.86	0.31
27. I want to get away from myself. (e)	0.80	0.05
28. I feel powerless to change myself. (e)	0.78	0.49
29. I would like to escape from my thoughts and feelings. (e)	0.83	0.33
30. I feel trapped inside myself. (e)	0.79	0.44
31. I would like to get away from who I am and start again. (e)	0.88	0.33
32. I feel I’m in a deep hole I can’t get out of. (e)	0.39	0.42

#### Confirmatory factor analysis (CFA) of the one factor model

We used a CFA to test whether a one factor model fits the data. All 32 items were allowed to load on one single factor. The one factor model showed a good fit to the data (CFI = 0.999, RMSEA = 0.014, SRMR = 0.056).

#### Exploratory graph analysis

The bootstrapped EGA of the online sample identified four dimensions.

Thirteen items of the defeat scale clustered together, and assessed all forms of feeling defeated. Three items from the defeat scale items formed a different cluster: *I feel that I am a successful person, I feel I am basically a winner, I feel able to deal with whatever life throws at me.*

The entrapment items were divided in two clusters: 10 items on internal entrapment *(*e.g.*, I want to get away from myself, I would like to escape my thoughts and feelings)* and six items on external entrapment *(*e.g.*, I would like to escape from my situation, I am in a relationship I cannot escape from).*

#### Confirmatory factor analysis of the four factor structure

To test whether the four-factor structure as proposed by the bootstrapped EGA fitted the data, we estimated a CFA by specifying the four proposed factors in lavaan: Factor one (defeat) = items 1, 2, 5 to 8 and 10 to 16, factor two (winner) = item 3, 4 and 9, factor three (external entrapment) = items 17 to 26, factor four (internal entrapment) = items 28 to 32. Factors were allowed to correlate. The four-factor structure fitted the data well (CFA = 1.00, RMSEA = 0.00, SRMR = .04). When using an ANOVA to compare the four factor model with the one factor model, the one factor model was preferred (*p* < 0.001).

### Results in the clinical sample (*N* = 147)

#### Exploratory factor analysis (EFA)

In the clinical sample, 40% of the variance was explained by the first factor, and 10% by the second factor. Although three factors had an eigenvalue > 1, the elbow of the scree plot appeared after factor one. Within the one factor model, all items except 4 items loaded above 0.4 indicating most items load at least reasonably well on the one factor (Table 2).

#### Confirmatory factor analysis (CFA) of the one factor model

As with the online sample, the one factor model showed a good fit to the data (CFI = 0.983, RMSEA = 0.049, SRMR = 0.098). However, lavaan indicated that the number of observations was too small to reliably compute the CFA.

#### Exploratory graph analysis

The bootstrapped EGA resulted in a three-factor solution. The same two dimensions within the entrapment items (internal and external entrapment) were identified as in the online sample, with only one item (*I am in a situation I feel trapped in*) clustered differently. Within the online sample, the item “*I am in a situation I feel trapped in*” was added to the external entrapment community as suggested by the original theory. Within the clinical sample, it was part of the internal entrapment community. All 16 defeat items were labelled as being part of one cluster. A warning was given that the correlation matrix was not a positive definite, probably related to the high number of parameters estimated in relation to the sample size. Therefore, results must be interpreted with caution.

#### Confirmatory factor analysis (CFA) of the three factor structure

The proposed three factors were used in lavaan to test the fit of the proposed structure to the data. Factor one (defeat) contained items 1 to 16, factor two (external entrapment) items 18 to 26 and factor three (internal entrapment) item 17, and items 27 to 32. The three factors were allowed to correlate. A CFA on the three factor model revealed a good fit to the data (CFI = 1.00, RMSEA = 0.00, SRMR = 0.067). Again, lavaan indicated that the number of observations was too small to reliably calculate the CFA. When comparing the CFA of the three factor model with the one factor model as proposed by the EFA, the one factor model was preferred (*p* < 0.001).

## Discussion

Recent studies indicate that defeat and entrapment are two sides of the same medal. In this study, we used novel network based techniques to re-estimate the number of clusters within the 32 items that make up the defeat and entrapment scales. When analyzing data from an online and a clinical sample, we found that the defeat and entrapment items did not cluster into one factor (cf. [24, 26]), but rather into distinct, yet, highly related factors. Even more, within the online sample, the walktrap algorithm clustered the first ten items that are related to external entrapment as indicated in the manual of the entrapment scale into a separate cluster, the six items that were originally constructed to assess internal entrapment [1] formed a second cluster within the entrapment items.

Similar results were found for the clinical sample, with one item of the internal entrapment scale (*I am in a situation I feel trapped in*) being added to the external cluster.

Regarding the concept of defeat, in the online sample, 13 of the 16 items formed one cluster. The three items that are positively framed seemed to form a separate cluster, indicating that participants tend to validate these items not merely as reversely coded defeat items. In factor analytic analyses this phenomenon is called “wording effect”, which means that the positive or negative formulation of items influences the interpretation of these items and the response of the participants. In its consequence, it has an effect on the factorial structure. This effect of the wording on the factorial structure has been confirmed in different studies on the dimensionality of other psychometric instruments like the Rosenberg Self-Esteem Scale and the Life-Orientation-Test [45–48]. In the clinical sample, all 16 defeat items were grouped into one dimension, thus, a wording effect was not detected here.

### Implications for research and clinical practice

The outcomes of our exploratory and confirmatory analysis were highly comparable with the analysis of Griffith et al. [26]. One factor explained most of the variance and all items loaded well on the one factor. The graph analysis showed that the items belonged to subtler sub-dimensions. The entrapment items can be thought of as highly related but different from the defeat items, and might even consist of two subscales, internal and external entrapment. Still, when comparing the CFAs, the one factor model as proposed by the EFA was preferred above the three or the four factor structure as proposed by the EGA on the clinical or the online sample. Known as Occam’s Razor, when the fit of two models are highly comparable, the simplest model is preferred [49]. However, no clear answer can be provided what can be considered as the best number of dimensions. The choice of the optimal number of dimensions should be a tradeoff between theory, clinical usefulness, model complexity and fit. The original theory by Gilbert and Allan [1] and more contemporary theories incorporating defeat and entrapment such as the IMV model [12, 13] propose that defeat and entrapment are different constructs. The original theory [1] even suggests that entrapment consists of the sub-dimensions internal and external entrapment. Although replication in larger (clinical) samples is needed our data appear to be in line with these theories and should be interpreted in the light of their assumptions.

On that note, with regard to the Short Defeat and Entrapment Scale of Griffiths et al. [26], we think that a shorter version of the DS and ES can be beneficial for clinical practice. However, it seems that the techniques used to come to the short scale were not able to deal with the highly correlated constructs of defeat and entrapment and their sub-dimensions. We therefore advise researchers not to simply use the short scale, but to select items from a theoretical and clinical perspective. The results of the present investigation may help clinicians and researchers in picking the appropriate items. Still, it is possible that the most informative items will differ from study to study, sample to sample, and perhaps even from patient to patient [50, 51].

From a clinical perspective, it is important to note that the present results support most theoretical clinical models incorporating defeat and entrapment as separate constructs. Most prominent, the IMV [12, 13] proposes that patients in a suicidal crisis develop states of defeat and entrapment consecutively when progressing from suicide ideation to suicide plans and behavior.

Practically, clinicians should be aware that feeling defeated and feeling entrapped most of the times coincide, but, most importantly, can also develop independently from each other. Each of these constructs may entail heightened risk for symptom deterioration or, when referring to suicidology, exacerbate the risk for the development of suicidal thoughts and behavior. Thus, both constructs should be part of a comprehensive risk assessment and should be born in mind when conceptualizing treatment for patients at risk for suicide.

### Limitations

An important limitation is the small sample size of the clinical sample. Although power analysis within network psychometrics is still being discussed, it is argued that to estimate a network of 32 items, at least 32 * (32–1/2) = 496 participants are needed. By this standard, even the online sample was marginally too small. By using bootstrap techniques to estimate our network, we have applied the current state of the art techniques to estimate stable networks. Although rather small, we added the clinical sample to further validate the findings of the online sample and to stimulate researchers with access to a larger clinical sample to use the same techniques and re-run our analyses. To facilitate this, a short online description of the used code was made: https://derekdebeurs.shinyapps.io/Online_code/#section-introduction.

Additionally, the number of dimensions within the online sample differed from the number of dimensions of the clinical sample. Validation in larger samples should investigate whether this differences are real differences between samples or the result of the difference in sample size. Finally, network psychometrics is a rapidly developing field, so new techniques to estimate communities using networks might be developed in the near future.

## Conclusions

In sum, regarding the latent factor structure of defeat and entrapment, we found that there is more than meets the eye (or traditional factor analysis can reveal). Results suggest, that they are separate but highly correlated constructs and that entrapment consists of the sub-dimensions internal and external entrapment. Traditional analyses tend to underestimate the number of dimensions in highly correlated data [26]. As psychological constructs tend to often be highly correlated, the application of novel graph techniques can improve our understanding of underlying dimensions of instruments assessing psychological constructs.
